# Pneumothorax After a Fall in a Healthy Adolescent Athlete

**DOI:** 10.7759/cureus.26293

**Published:** 2022-06-24

**Authors:** Tristin Latty, Rebekah J Soto, Carlos A Arango

**Affiliations:** 1 Pediatrics, University of Florida, Gainesville, USA; 2 Pediatrics, University of Florida, Jacksonville, USA

**Keywords:** risk factors for pneumothorax, pediatrics patient, pediatrics, blunt thoracic trauma, pneumothorax (ptx)

## Abstract

A pneumothorax is a pathological collection of air in the pleural space within the thoracic cavity. Pneumothoraxes are classified by the underlying etiology as spontaneous or traumatic, with spontaneous pneumothoraxes being further categorized into primary and secondary causes. Management historically involved admission with possible administration of oxygen or chest tube placements based on severity. We herein describe a pediatric ­case of a likely blunt-force traumatic pneumothorax after a fall, successfully managed conservatively in the outpatient setting. The case highlights an acceleration-deceleration blunt trauma caused during a tackle at a football game and the importance of the clinical presentation, physical exam, and confirmation with a chest X-ray.

## Introduction

A pneumothorax is a pathological collection of air in the pleural space within the thoracic cavity. Pneumothoraxes are classified by the underlying etiology as spontaneous or traumatic, with spontaneous pneumothoraxes being further categorized into primary and secondary causes.

## Case presentation

A 17-year-old male presented to our clinic with his mother complaining of chest pain for the prior two days. The patient reported the chest pain started the day prior to the presentation after falling on his back while he was playing football for his high school. He was attempting to catch a football when he fell directly on his back after being tackled, and he stated, "the wind was knocked out of me." He was able to get back into the game after he caught his breath, but reported that he continued to feel as though there was "something inside of my chest." The patient took 400 mg ibuprofen tablets twice daily with no improvement in his pain. He denied hitting his head or neck and having no loss of consciousness. No fever, visual disturbances, dizziness, headache, neck pain, shortness of breath, abdominal pain, nausea/vomiting/diarrhea, or any other symptoms were reported.

The patient’s medical history was unremarkable, and he did not take any chronic medications or supplements. The patient’s family history was unknown as he was adopted. The patient did not have a history of surgeries or hospitalizations. Upon further questioning privately, the patient denied sexual activity. He denied smoking, vaping, drinking, or using illicit drugs as he takes his athletic responsibilities seriously. The patient was up-to-date on his vaccinations and he denied having any drug allergies.

In the office, the patient’s vitals were stable, and he was afebrile with a blood pressure of 115/76, a heart rate of 65, and O2 saturation of 100%. The patient’s height and weight were 158 lbs (67th percentile) and 5’10" (62nd percentile), respectively. On physical exam, the patient was alert, well developed, well-nourished, and not in acute distress. The head was normo-cephalic and atraumatic. Pupils were equal, round, and reactive to light and accommodation with normal conjunctiva and alignment. The neck was supple with no masses, thyromegaly, adenopathy, and a full range of motion. On palpation of the patient’s supraclavicular area, crepitus was noted on the patient’s right side. Examination of the patient’s lungs revealed a slight decrease in breath sounds around the right middle lower lobe with increased fremitus around the same area. There were no wheezes, rales, or rhonchi.

**Figure 1:**
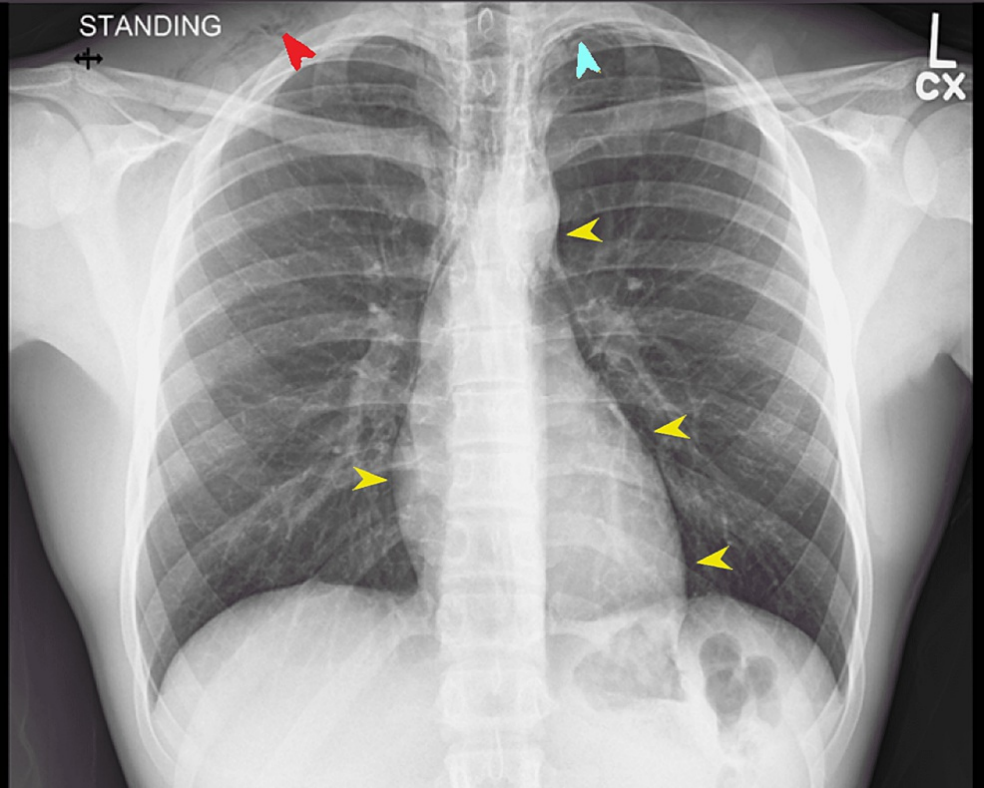
AP chest X-ray Chest X-ray demonstrates pneumomediastinum (yellow arrows), with an apical pneumothorax (blue arrow), and free air in the supraclavicular soft tissue (red arrow)

**Figure 2:**
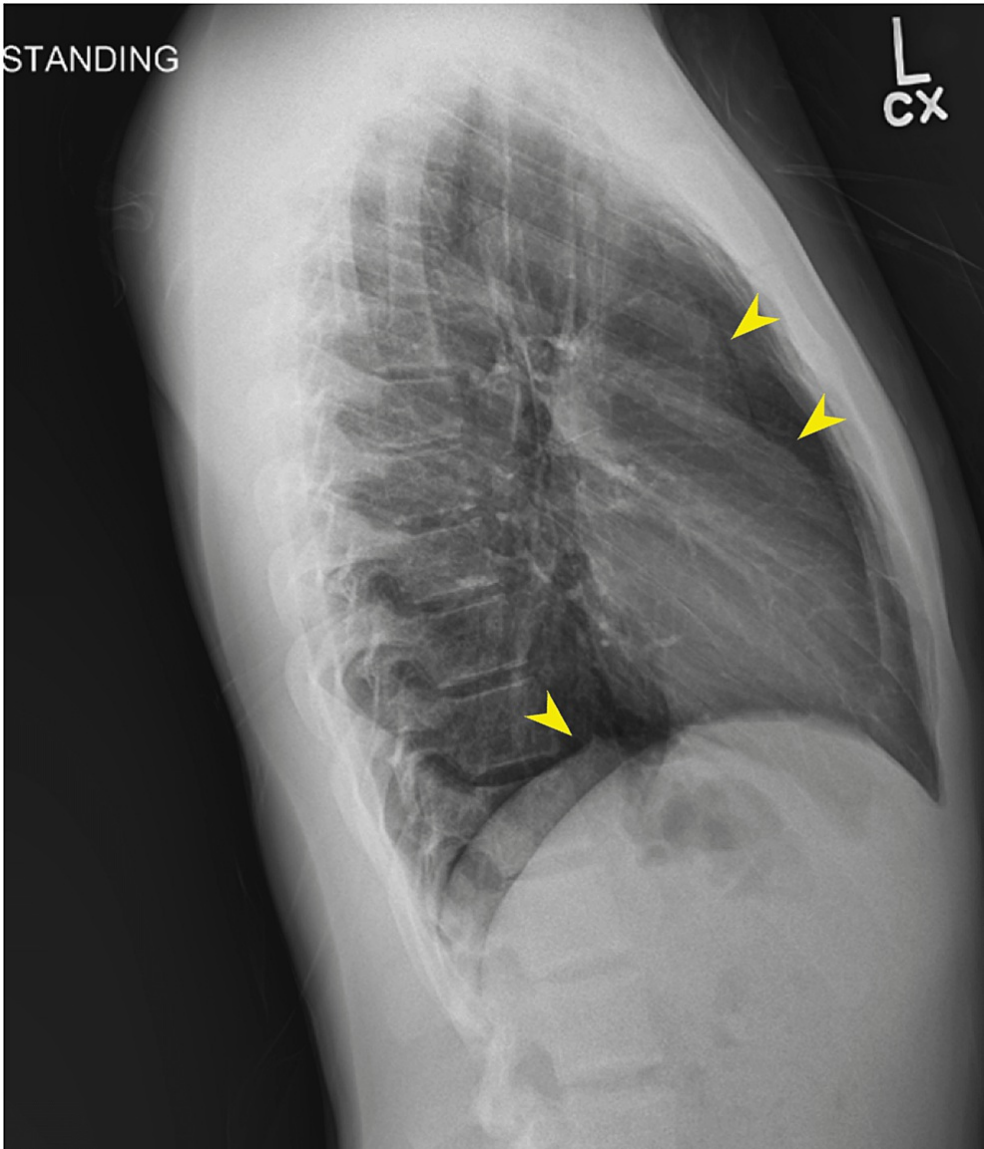
Lateral chest X-ray Yellow arrows: pneumomediastinum

A cardiac exam revealed a regular rate and normal rhythm with a normal S1 and S2, with no murmurs, rubs, or gallops. Dorsalis pedis and radial pulses were 2+. The rest of the physical exam was unremarkable. An AP and lateral chest X-ray were ordered STAT and a wet read was requested (Figures 1-2).

The images (Figures 1-2) were interpreted as a trace of right apical pneumothorax and a small volume mediastinum. The patient was notified of the findings and instructed to avoid all physical activities for the time being. Given that the patient was stable and relatively asymptomatic, the patient was instructed to follow up in the office seven days after repeat imaging. Instructions for when to present themselves to the emergency department were also given.

**Figure 3:**
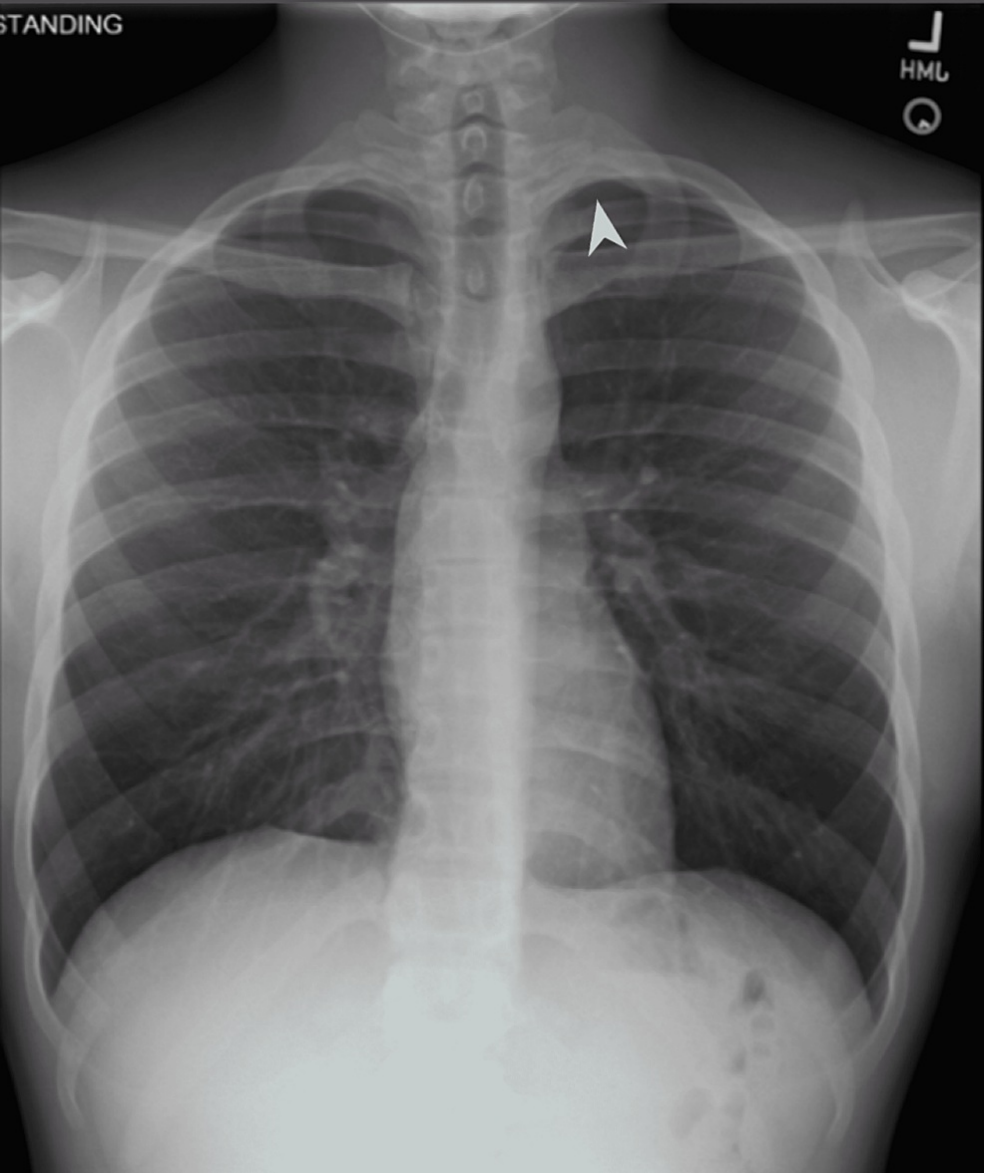
One-week follow up Residual small right apical hypo-density

**Figure 4:**
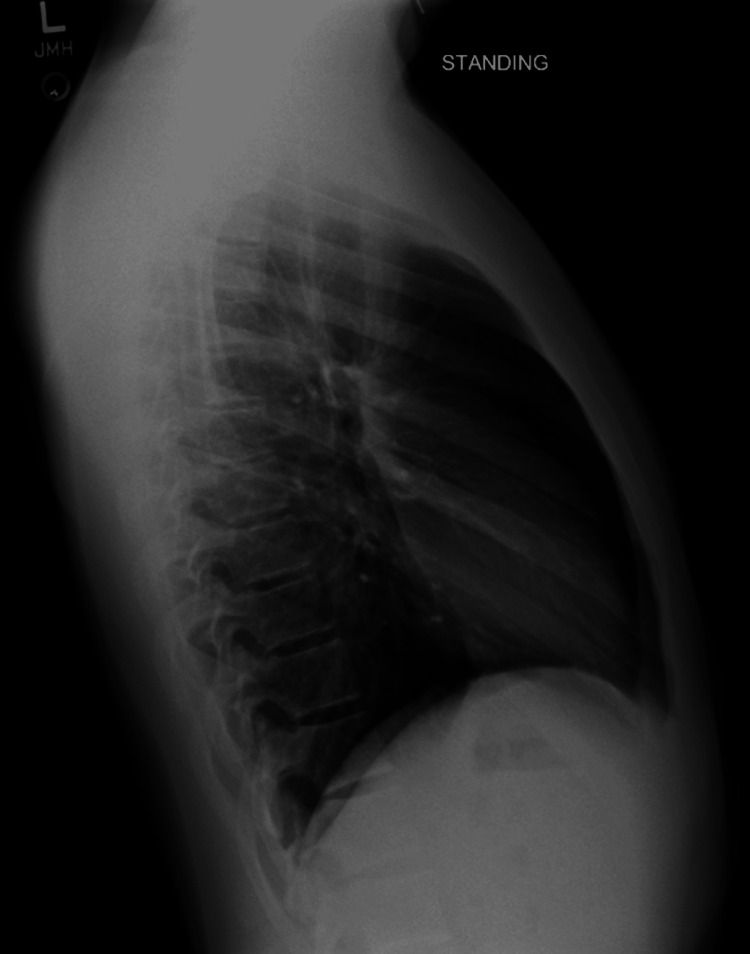
Lateral chest X-ray: one week follow up Normal reading

The patient presented to the clinic the following week where repeat imaging (Figures 3-4) demonstrated significant improvement in his pneumothorax with a residual small left apical hypo-density. The patient no longer complained of chest pain or shortness of breath and his physical exam was back to baseline with no palpable supraclavicular crepitus. The patient was eager to resume sports, but he was instructed to refrain from all contact sports for at least six weeks, including long jumps and diving.

## Discussion

We present the case of a previously healthy 17-year-old male patient with a symptomatic pneumothorax that developed after a fall while playing football. Our patient’s pneumothorax and associated symptoms spontaneously resolved with conservative management. While pneumothorax can be the cause of chest pain in an otherwise healthy and athletic male adolescent, identification of pneumothorax in this population requires a high index of suspicion from the treating physician and thorough patient history and physical examination.

A pneumothorax describes an abnormal collection of air in the pleural space between the visceral and parietal pleura in the thoracic cavity. Although there is a paucity of data on the pediatric population, one multisite study estimates the overall incidence to be on average 3.41/100,000 in children aged 0 to 17 years, with the incidence being higher in males in comparison to females (5.28 vs 1.27/100,000), adolescents versus younger children less than 10 years of age (>80% vs <7%), and those with lower BMI [1,2]. 

Understanding the underlying etiology of a pneumothorax may help guide management and workup. Therefore, it is important to classify a pneumothorax as traumatic or spontaneous based on the patient's history. Furthermore, a spontaneous pneumothorax should be classified as primary or secondary. A primary spontaneous pneumothorax occurs in patients without an underlying disease, whereas a secondary spontaneous pneumothorax occurs in patients with diseases affecting the lung parenchyma, such as pneumonia, cystic fibrosis, asthma, or connective tissue diseases. Treatment of underlying disease needs to be addressed after initial management of the secondary spontaneous pneumothorax to prevent recurrence [3]. Though not a factor in our case, vaping and smoking are two potential risk factors for pneumothorax that must be addressed, especially in adolescents. The use of vaping devices and cannabis has been associated with lung injury and pneumothorax in otherwise healthy young adults [4,5].

Our patient most likely had a blunt force traumatic pneumothorax given the onset of his symptoms after falling on his back forcefully; however, a primary spontaneous pneumothorax cannot be excluded. A more typical presentation of a traumatic pneumothorax in a pediatric patient would likely involve a greater degree of blunt force trauma, such as a motor vehicle collision, bicycle accident, or fall from a height, thus causing severe symptoms and may be accompanied by additional radiological findings including rib fractures or pulmonary contusions [6,7].

The diagnosis of traumatic pneumothorax was made after a thorough physical exam, history, and imaging were completed. Possible contributing risk factors including smoking, vaping, recent illness, substance use, and underlying disease were considered and ruled out, leading to the diagnosis of blunt force trauma as the cause of his pneumothorax.

Currently, guidelines to date regarding the treatment of blunt force traumatic pneumothoraxes in the pediatric patient population, and management is guided by existing guidelines in adults. Current guidelines that are commonly used for the management of spontaneous pneumothorax in adults include those proposed by the American College of Chest Physicians (ACCP), the European Respiratory Society (ERS), and the British Thoracic Society (BTS). The ERS and BTS guidelines emphasize the importance of clinical symptoms over the size of the pneumothorax to guide care, while the ACCP recommends the placement of an intercostal drain for a pneumothorax larger than 20% of the hemithorax, regardless of symptoms. The use of needle aspiration, chest tube insertion, oxygen administration, or surgical innovations is addressed in these guidelines [8,9].

Current guidelines suggest treating symptomatology rather than the size of the pneumothorax. If the size is small and the patient is asymptomatic, then a conservative approach is the best choice. Simple needle aspiration may be effective in up to 70% of cases if the patient has been hospitalized and oxygen therapy has not produced symptom relief. Chest tube insertion is needed when needle aspiration has failed or if significant symptoms develop [10].

Considering our patient was clinically stable with minimal symptoms, easy access to care, and a relatively small pneumothorax on imaging, conservative management with close outpatient follow-up was recommended. The patient was counseled regarding signs and symptoms of worsening pneumothorax, including worsening chest pain, shortness of breath, and pain with breathing or respiratory distress.

Our patient had substantial improvement both clinically and on imaging at one-week follow-up; therefore, the patient was discharged with contact sports precautions. Given that a primary spontaneous pneumothorax could not be excluded, the patient was advised that there is a risk of recurrence, especially in pediatric populations. Recurrence of a pneumothorax may suggest an underlying cause, and further workup may be indicated at that time. At the time of this publication, the patient remains asymptomatic without recurrent pneumothorax.

## Conclusions

We present the case of a healthy male adolescent who presented to the outpatient clinic with a history of chest pain that began after a fall at football practice two days prior. The diagnosis of traumatic pneumothorax was made after a thorough physical exam, history, and imaging were completed. Possible contributing risk factors and underlying medical conditions were considered and ruled out, leading to the diagnosis of blunt force trauma as the cause of his pneumothorax. While athletic activities and injuries are common in the adolescent population, to the best of our knowledge, this is the first case report of its kind discussing the development of a pneumothorax in a healthy adolescent after a forceful fall at football practice.

## References

[1] Dotson K, Timm N, Gittelman M (2012). Is spontaneous pneumothorax really a pediatric problem? A national perspective. Pediatr Emerg Care.

[2] Kaslow J, Bickel S, Wiesenauer C, Eid N, Morton R (2018). Pediatric Allergy, Immunology, and Pulmonology.

[3] Yousuf S, Cardenas S, Rezaee F (2021). Pediatric pneumothorax: case studies and review of current literature. Respir Med Case Rep.

[4] Cheng YL, Huang TW, Lin CK, Lee SC, Tzao C, Chen JC, Chang H (2009). The impact of smoking in primary spontaneous pneumothorax. J Thorac Cardiovasc Surg.

[5] Borchert DH, Kelm H, Morean M, Tannapfel A (2021). Reporting of pneumothorax in association with vaping devices and electronic cigarettes. BMJ Case Rep.

[6] Nakayama DK, Ramenofsky ML, Rowe MI (1989). Chest injuries in childhood. Ann Surg.

[7] Dogrul BN, Kiliccalan I, Asci ES, Peker SC (2020). Blunt trauma related chest wall and pulmonary injuries: An overview. Chin J Traumatol.

[8] MacDuff A, Arnold A, Harvey J (2010). Management of spontaneous pneumothorax: British Thoracic Society Pleural Disease Guideline 2010. Thorax.

[9] Tschopp JM, Bintcliffe O, Astoul P (2015). ERS task force statement: diagnosis and treatment of primary spontaneous pneumothorax. Eur Respir J.

[10] DeMaio A, Semaan R (2021). Management of pneumothorax. Clin Chest Med.

